# RAGE deficiency does not affect non-alcoholic steatohepatitis and atherosclerosis in Western type diet-fed Ldlr^**−/−**^ mice

**DOI:** 10.1038/s41598-018-33661-y

**Published:** 2018-10-15

**Authors:** Mitchell Bijnen, Nicky Beelen, Suzan Wetzels, José van de Gaar, Maria Vroomen, Erwin Wijnands, Jean L. Scheijen, Marjo P. H van de Waarenburg, Marion J. Gijbels, Jack P. Cleutjens, Erik A. L. Biessen, Coen D. A. Stehouwer, Casper G. Schalkwijk, Kristiaan Wouters

**Affiliations:** 1 Department of Internal Medicine, MUMC, Maastricht, The Netherlands; 2 CARIM, MUMC, Maastricht, The Netherlands; 3 Department of Immunology and Biochemistry, Biomedical Research Institute, Hasselt University, Hasselt, Belgium; 4 Department of Pathology, MUMC, Maastricht, The Netherlands; 5 Department of Molecular Genetics, MUMC, Maastricht, The Netherlands; 6 Department of Medical Biochemistry, Experimental Vascular Biology, AMC, Amsterdam, The Netherlands

## Abstract

Non-alcoholic fatty liver disease is a spectrum of liver diseases ranging from steatosis only to non-alcoholic steatohepatitis (NASH). The latter is characterized by hepatic inflammation, which increases the risk of cardiovascular disease. It is poorly understood which factors contribute to the onset of hepatic inflammation characterizing the progression from steatosis to NASH. Previously, we demonstrated increased advanced glycation endproducts (AGEs) in the livers of NASH patients. We hypothesise that AGEs play a key role in NASH development by activating their proinflammatory receptor, RAGE. RAGE-deficient mice and wildtype littermates, both on Ldlr^−/−^ background, were fed a Western type diet (WTD) for 3 or 12 weeks. Flow cytometry, histology, gene expression and AGE measurements were performed to evaluate the effects of RAGE deficiency. RAGE-deficient mice displayed reduced weight gain and visceral fat expansion compared to control mice. No difference in adipose tissue inflammation was observed between groups. RAGE deficiency did not affect WTD-induced monocytosis, circulating lipids or hepatic steatosis. WTD-induced hepatic neutrophil and macrophage accumulation and atherosclerotic plaque development was comparable between control and RAGE-deficient mice. No difference in AGE levels was observed. RAGE does not seem to play a major role in the development of NASH or atherosclerosis in a hyperlipidemic mouse model.

## Introduction

Non-alcoholic fatty liver disease (NAFLD) is the most common liver disease, affecting 20–30% of the general population of the western world^1^. Its prevalence has risen in tandem with obesity, which is a key risk factor for NAFLD. Obesity increases the risk of developing NAFLD 3.5-fold and was shown to contribute to hepatic inflammation^2–5^. This higher risk is attributed to alterations in glucose and lipid metabolism as well as lipid-induced chronic systemic inflammation^4^. A more advanced and severe form of NAFLD is non-alcoholic steatohepatitis (NASH), which is characterized by hepatic lipid accumulation (steatosis) in combination with hepatic inflammation^3^. NASH can cause extensive hepatic fibrosis and cirrhosis, leaving only liver transplantation as an option for these patients^6^. In addition, NASH is linked to cardiovascular disease (CVD) with over five times as many NASH patients dying from CVD instead of liver-related causes^6,7^. Furthermore, long-term survival of CVD-related diseases is lower in NASH patients compared to NAFLD patients with steatosis only^7^. Therefore, it is of great importance to identify the triggers involved in the progression of simple hepatic steatosis to NASH.

Liver-resident macrophages, the Kupffer cells, play an important role in liver injury, pathogen defence and thus hepatic inflammation. Many factors may contribute to hepatic inflammation and Kupffer cell activation, such as an abundance of liver fat and cholesterol, circulating inflammatory cytokines, oxidative stress and advanced glycation endproducts (AGEs)^8,9^. AGEs are formed by the reaction of reduced sugars with amino groups of proteins and their formation is a consequence of normal metabolism^10^. Hyperlipidaemia, hyperglycaemia, and (inflammation induced) oxidative stress, which are prominently present in the livers of NAFLD patients, contribute to the formation of AGEs. Indeed, AGE accumulation in plasma and tissue is associated with obesity, diabetes and atherosclerosis, an important cause of CVD^11–13^. We previously showed that N^ε^-(carboxymethyl)lysine (CML), an important AGE, accumulates in human livers and is linked to NASH^9^. In addition, we demonstrated that CML exerts proinflammatory effects on hepatocytes via the receptor of AGEs, RAGE^9^. RAGE is a cell surface receptor capable of recognizing multiple ligands (AGEs, HMGB1, S100 proteins), it is expressed on hepatocytes, stellate cells, lymphocytes, endothelial cells, monocytes and macrophages including Kupffer cells^14,15^. Therefore, AGE-induced activation of RAGE potentially contributes to the onset of inflammation in NASH. Previous studies have shown reduced inflammation, reduced atherosclerotic plaque size and improvement of insulin sensitivity in RAGE-deficient mice^16–18^. In the current study, we investigated whether RAGE is causally linked to hepatic inflammation and atherosclerosis development during Western type diet (WTD)-induced NASH development by comparing control Ldlr^−/−^ mice with RAGE-deficient Ldlr^−/−^ mice.

## Materials and Methods

### Animal study

Female 9–11 week old C57BL/6 Ldlr^−/−^ (henceforth referred to as WT) and littermate Ldlr^−/−^RAGE^−/−^ (henceforth referred to as KO) mice were fed a WTD (21% milk butter, 0.2% cholesterol, 46% carbohydrates (of which 40,5% sucrose) and 17% casein; SDSdiets #824171) for 3 (n = 7) or 12 weeks (n = 11–12) after which they were sacrificed using CO_2_/O_2_ inhalation followed by exsanguination via cardiac puncture. WTD feeding strongly and rapidly induces hepatic steatosis and inflammation, main hallmarks of NASH, and atherosclerosis in Ldlr^−/−^ mice^19,20^. Considering the abundance of previous studies using this model show that this model displays hepatic steatosis and inflammation, we only investigated the role of RAGE in WTD-fed mice^5,19–22^. Standard chow-fed mice, which develop neither atherosclerosis nor NASH, were therefore not included due to ethical reasons^5,19–22^. The RAGE^−/−^ mice used for breeding were a generous gift from Prof. Dr. P. P. Nawroth (Heidelberg University). All performed experiments were approved by the Animal Experiments Committee of Maastricht University and in compliance with the relevant guidelines from the Directive 2010/63/EU of the European Parliament on the protection of animals used for scientific purposes.

### Flow cytometry

During the study, blood was taken from the hind limb using EDTA lined Microvette tubes (Sarstedt) and at sacrifice by cardiac puncture. Blood was transferred to a Trucount absolute count tube (BD) containing FC-receptor block (anti-CD16/CD32) before adding a cocktail of antibodies (Suppl. Table 1). Hereafter, erythrocytes were lysed using lysisbuffer (8.4 g/L NH_4_CL and 0.84 g/L NaHCO_3_ in H_2_O, pH 7.4) before measurement. Flow cytometry was performed using a BD FACSCANTO II running FACS Diva 8.0.1 software which was also used for all analyses. The FACS gating strategy can be found in the Supplementary Materials (Suppl. Fig. 1).

### RNA Isolation, cDNA Synthesis and qRT-PCR

RNA was isolated from liver, visceral fat and aortic arch tissue using Trizol reagent (Ambion) before cDNA synthesis using the iScript cDNA synthesis kit (170–8891; Bio-Rad, Hercules, USA) following manufacturer’s instructions. Gene expression was determined using IQ SensiMix SYBR master mix (Bioline, London, UK) on a CFX96 Touch with CFX manager software (Biorad). The geometric mean of two reference genes, Cyclophillin and Beta2-microglobulin, was used as reference and the ΔΔCT method was employed to calculate expression levels^23^. Primer sequences are given in Supplementary Table 2.

### Histology

Frozen liver sections (7 µm) were stained using antibodies against macrophages (rat anti-mouse F4/80, clone Cl:A3-1; Acris antibodies) and neutrophils (custom antibody; clone NIMP-R14) as previously described^20,24^. As negative controls, the primary antibody was omitted. Photographs were captured using a Zeiss microscope (Axioskop 40) with a Jenoptik camera and Progress Capture Pro 2.8.8 software. Neutrophils were counted in 6 microscopical views (200x magnification) of the liver while hepatic macrophage content was quantified as percentage of F4/80^+^ pixels of the total area using Photoshop CS3 (v10.0). Liver (4 µm) and adipose tissue (7 µm) paraffin sections were stained for Haematoxylin and Eosin (H&E; Sigma-Aldrich) and photos were taken (200x magnification) using the setup described above. General hepatic inflammation (immune cell count and clustering, hepatocyte injury) was scored in masked fashion by an experienced mouse pathologist using the H&E-stained sections. Adipose tissue sections were analysed in a blinded manner to determine adipocyte cell size using computerized morphometry (Leica QWin V3, Cambridge, UK). Aortic roots were cryosectioned (7 µm) and stained with H&E or Sirius Red for plaque area and collagen content quantification, respectively. These sections were analysed blindly using computerized morphometry to determine plaque size and collagen content (Leica QWin V3, Cambridge, UK). Atherosclerotic plaque phenotype was rated small, moderate or advanced based on fibroblasts, necrosis, foam cells, general inflammation, endothelial adhesion, granulocytes, adventitia influx and calcification by an experienced mouse pathologist using the H&E sections as previously described^25^.

### Hepatic and plasma cholesterol and triglyceride measurements

Livers were homogenized in 250 µl SET buffer (250 mM sucrose, 2 mM EDTA and 10 mM Tris) using a Mini-bead beater homogenizer (Biospec). Hepatic and plasma levels of cholesterol and triglycerides were measured using a colourimetric test (Cholesterol FS’10 and Triglycerides FS 5′ecoline, Diagnostic System GmbH, Holzheim, Germany) as described previously^26^. Liver lipid levels were corrected for protein content by performing a BCA assay (BCA kit, Sigma-Aldrich, Germany) according to the manufacturer’s instructions.

### α-dicarbonyls, AGE and glyoxalase measurements

Livers were homogenized in SET buffer as described above. Methylglyoxal (MGO), glyoxal (GO), 3-deoxyglucosone (3-DG) and free N^ε^-(carboxymethyl)lysine (CML), N^ε^-(1-carboxyethyl)lysine (CEL), and N^δ^-(5-hydro-5-methyl-4-imidazolon-2-yl)-ornithine (MG-H1) were analysed in plasma or liver and adipose tissue homogenates by ultra-performance liquid chromatography tandem mass spectrometry (UPLC MS/MS) as previously described^27,28^.

Glyoxalase-1 (Glo-1) activity was measured by spectrophotometry by determining the increase in absorbance at 240 nm due to S-d-lactoyl-glutathione formation as described by McLellan *et al*.^29^.

### Bone marrow-derived macrophages (BMDMs) culturing

Femur and tibia were obtained from C57BL/6 Ldlr^−/−^ (WT) and littermate Ldlr^−/−^RAGE^−/−^ (KO) mice and bone marrow was flushed using PBS. The bone marrow was filtered into a single cell suspension before centrifuging and resuspending the cells in RPMI 1640 (Greiner 31870-074) medium containing 15% L929 cell conditioned medium, 10% Fetal Calf Serum (Hyclone) and 1% Glutamine pen/strep (Greiner). The cells were then plated and cultured for 7 days, necessary to allow complete differentiation to BMDMs, before discarding all non-adherent cells. BMDMs were incubated for 24 hours before stimulation with TNF (500 U/ml), Lipopolysaccharide (LPS; 10 ng/ml) or IFN-ɣ (100 U/ml) for 2 hours. After stimulation, BMDMs were washed with PBS and harvested using Tri reagent (Sigma-Aldrich) for RNA isolation. Experiments were performed in triplicate.

### Statistical Analysis

Data was tested for significance using a two-tailed Student’s T-test when comparing two groups or a one-way ANOVA with Tukey’s multiple comparison post-hoc test when comparing multiple groups. GraphPad Prism 5.01 (La Jolla California, USA) was used to perform all data analyses, all data was expressed as the mean ± SEM and were considered statistically significant at P ≤ 0.05.

## Results

### RAGE deficiency leads to reduced weight gain and visceral adipose tissue without affecting circulating lipids or monocytosis

**Figure 1:**
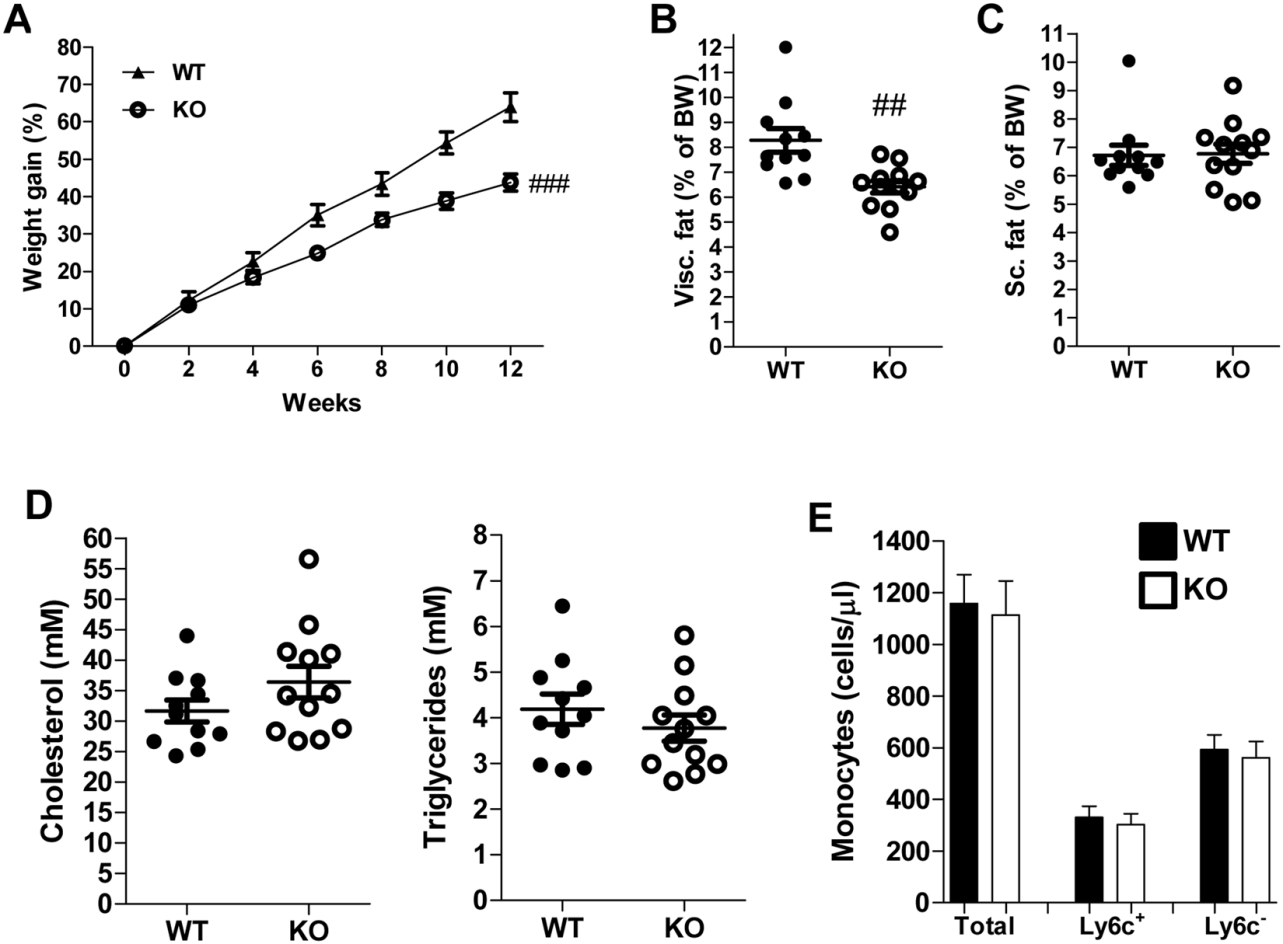
RAGE deficiency reduced weight gain and visceral adipose tissue but did not affect circulating lipids or monocytosis. (**A**–**C**) Bodyweight gain over time in the 12 weeks WTD-feeding experiment (**A**) and vAT (**B**) or sAT (**C**) as percentage of bodyweight. (**D**) Plasma cholesterol and triglyceride levels after 12 weeks of WTD feeding. (**E**) Total circulating monocyte levels and subdivision in Ly6c^+^ and Ly6c^−^ monocytes measured by flow cytometry and presented as cells/µl after 12 weeks of WTD feeding. All data are means ± SEM. ^##^P < 0.01, ^###^P < 0.001 vs WT. n = 11–12.

WTD-induced weight gain was markedly reduced in RAGE-deficient mice in comparison to control mice (Fig. 1A). Visceral adipose tissue (vAT) expansion was reduced in RAGE-deficient mice, while subcutaneous adipose tissue was not different between control mice and mice lacking RAGE (Fig. 1B,C). RAGE deficiency did not impact plasma cholesterol and triglyceride levels (Fig. 1D). WTD feeding triggers monocytosis increasing circulating monocyte levels^30^. However, flow cytometry revealed no difference between control and RAGE-deficient mice in total circulating monocytes or “proinflammatory” Ly6c^+^ and “patrolling” Ly6c^−^ monocyte subsets (Fig. 1E). All other circulating immune cells were comparable between the groups (Suppl. Table 3). Similar results were obtained after 3 weeks of WTD feeding (Suppl. Fig. 2A–C, Suppl. Table 4).

### RAGE deficiency does not affect hepatic steatosis or inflammation

**Figure 2:**
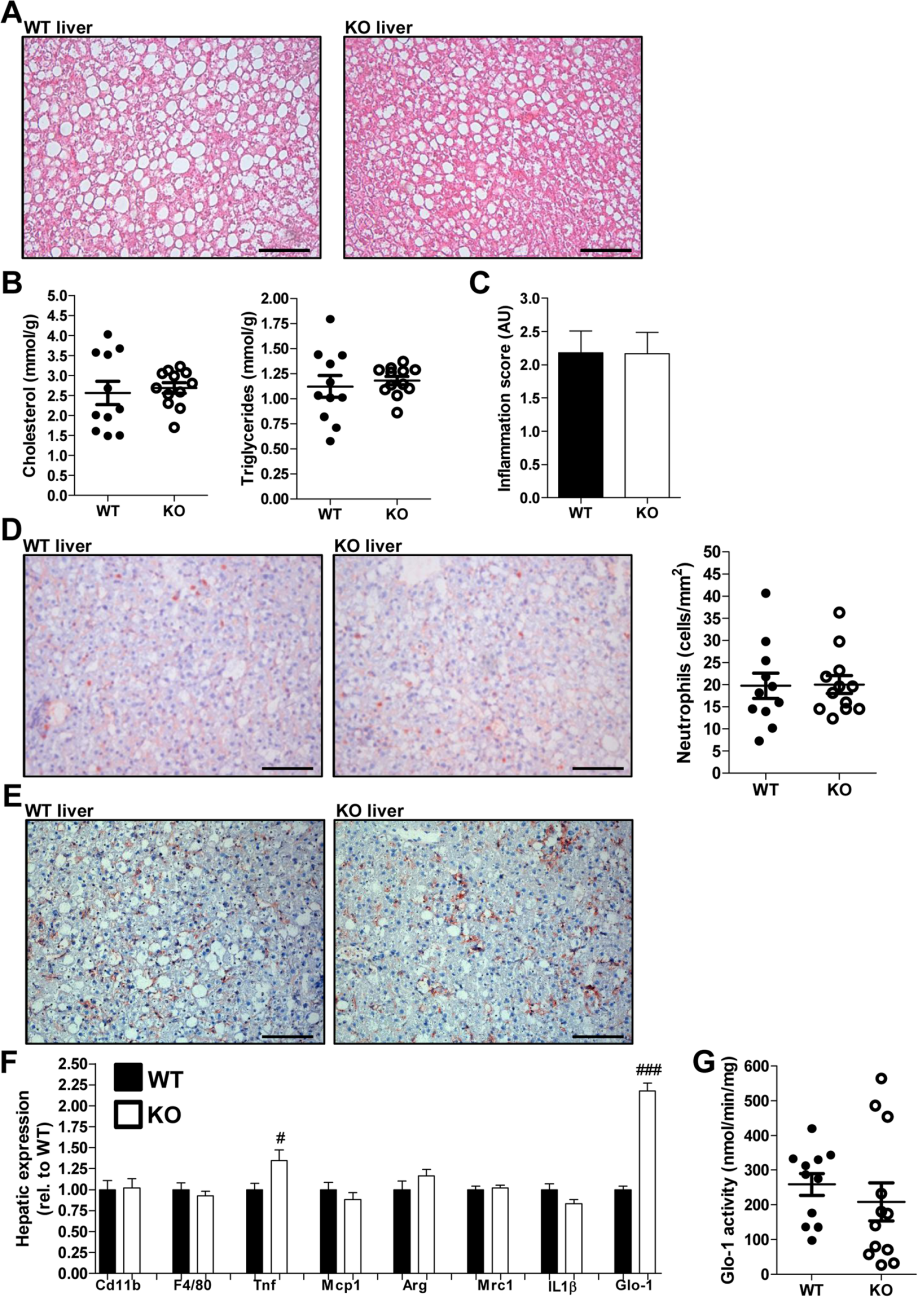
RAGE deficiency does not affect hepatic steatosis, neutrophil infiltration or macrophage accumulation. (**A**,**B**) Representative images of the liver stained with H&E (A; 200x magnification) and hepatic cholesterol and triglyceride levels after 12 weeks of WTD feeding (**B**). (**C**) Hepatic inflammation scored (1, 2, 3 or 4) by an experienced pathologist based on lobular inflammation and immune cell numbers after 12 weeks of WTD feeding. (**D**) Representative photos and quantification of immunohistochemical neutrophil staining (NIMP-R14 antibody; 200x magnification) in the liver after 12 weeks of WTD. (**E**) Representative images of hepatic macrophage staining (F4/80 antibody; 200x magnification) after 12 weeks of WTD feeding. (**F**) Total hepatic gene expression levels measured of inflammatory and immune cell specific markers after 12 weeks of WTD feeding. (**G**) Hepatic GLO-1 activity levels after 12 weeks of WTD feeding. All data are means ± SEM. ^#^P < 0.05, ^###^P < 0.001 vs WT. n = 11–12. Scalebars 100 µm.

WTD feeding strongly and rapidly induces hepatic steatosis and inflammation, main hallmarks of NASH, in Ldlr^−/−^ mice^19,20^. Indeed, after 12 weeks of WTD feeding, the liver was very steatotic and contained high, but similar levels of cholesterol and triglycerides in control and RAGE-deficient mice (Fig. 2A,B). RAGE deficiency was confirmed by measurement of RAGE hepatic gene expression, which was not detectable in the RAGE-deficient mice (Suppl. Fig. 3A). Lobular inflammation and immune cell presence was comparable in control and RAGE-deficient mice after 12 weeks of WTD (Fig. 2C). Hepatic neutrophil infiltration and macrophage accumulation was clearly present after WTD feeding, but similar in control and RAGE-deficient mice (Fig. 2D,E). Correspondingly, total liver *F4/80* (general macrophage marker) and *Cd11b* (marker of monocytes/macrophages and neutrophils) gene expression levels were comparable between groups (Fig. 2F). To further phenotype the accumulated macrophages, *Tnf* and *Mcp1* (proinflammatory markers) and *Arg1* and *Mrc1* (markers of anti-inflammatory macrophages) were measured. *Tnf* expression was significantly higher in RAGE-deficient mice compared to control mice while the other markers were not affected by RAGE deficiency. Expression of *Glo-1*, the rate-limiting enzyme in the detoxification of the major AGE precursor MGO^31^, was clearly higher in RAGE-deficient mice (Fig. 2F). However, Glo-1 activity was not affected by the absence of RAGE (Fig. 2G). After 3 weeks of WTD feeding, all expression levels were similar between groups except for *Glo-1* expression, which was again twice as high in the RAGE-deficient mice compared to the control mice (Suppl. Fig. 3B–F).

### RAGE does not affect visceral adipose tissue cell size, lipid metabolism or inflammation

RAGE deficiency affected weight gain and vAT expansion after 12 weeks of WTD feeding (Fig. 1A,B). This reduction in vAT expansion was not due to differences in adipocyte cell size (Suppl. Fig. 4A). Another potential explanation for the reduced weight gain could be an effect of RAGE on lipolysis or adipogenesis. Expression of *Lpl*, *Atgl* and *Hsl* (lipolysis related genes) was comparable between the groups (Suppl. Fig. 4B). Regarding adipogenesis, *Srebp1c* and *Cebp-α* gene expression was comparable between groups, while the RAGE-deficient mice had a slightly higher expression of *Ppar-γ* (Suppl. Fig. 4B).

Next, we investigated vAT inflammation. A general macrophage marker, F4/80, as well as a proinflammatory, Cd11c, and anti-inflammatory, Mrc1, macrophage marker were compared between control and RAGE-deficient mice. All these markers were similar between groups. In line, the expression of Mcp1 and Tnf, two inflammatory cytokines, was comparable between both groups of mice (Suppl. Fig. 4C).

### Atherosclerosis development is not affected by the presence of RAGE

**Figure 3:**
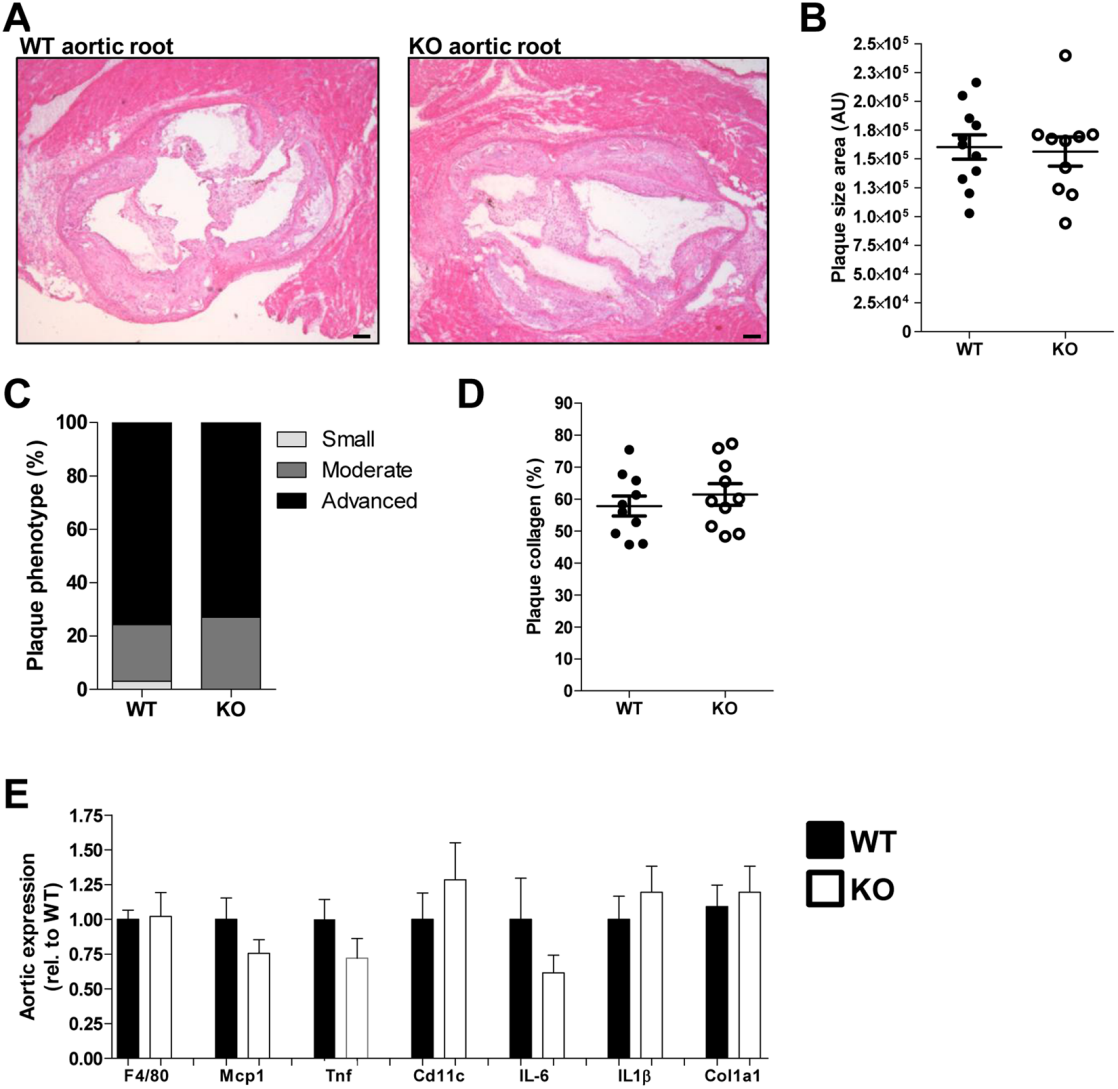
Atherosclerotic plaque size and inflammatory state is unaffected by RAGE. (**A**,**B**) Representative pictures (**A**) of H&E-stained aortic root plaques (40x magnification) and corresponding quantification of plaque size (**B**) after 12 weeks of WTD feeding. (**C**) Plaque phenotype score presented as percentage of each score. (**D**) Quantification of plaque collagen content presented as percentage of plaque size area. (**E**) Aortic arch gene expression levels of inflammatory and immune cell specific markers. All data are means ± SEM. n = 11–12. Scalebars 100 µm.

WTD feeding does not only induce hepatic steatosis and inflammation in Ldlr^−/−^ mice, but also atherosclerosis development, which was assessed after 12 weeks of diet. Both the control and RAGE-deficient mice developed significant plaques in the aortic root, but they did not differ in plaque size (Fig. 3A,B). In line, plaque phenotype (based on fibroblasts, necrosis, foam cells, general inflammation, endothelial adhesion, granulocytes, adventitia influx and calcification) and collagen content were comparable between both groups (Fig. 3C,D). *F4/80* expression in the aortic arch did not differ between control and RAGE-deficient mice, suggesting equal macrophage content. Proinflammatory markers, such as *Mcp1, Tnf*, *IL6*, *IL1β* and *Cd11c*, were also similar between control and RAGE-deficient mice. Aortic expression of Col1a1 was comparable between the groups as expected based on the plaque collagen content (Fig. 3E).

### RAGE does not contribute to inflammatory signalling in macrophages

**Figure 4:**
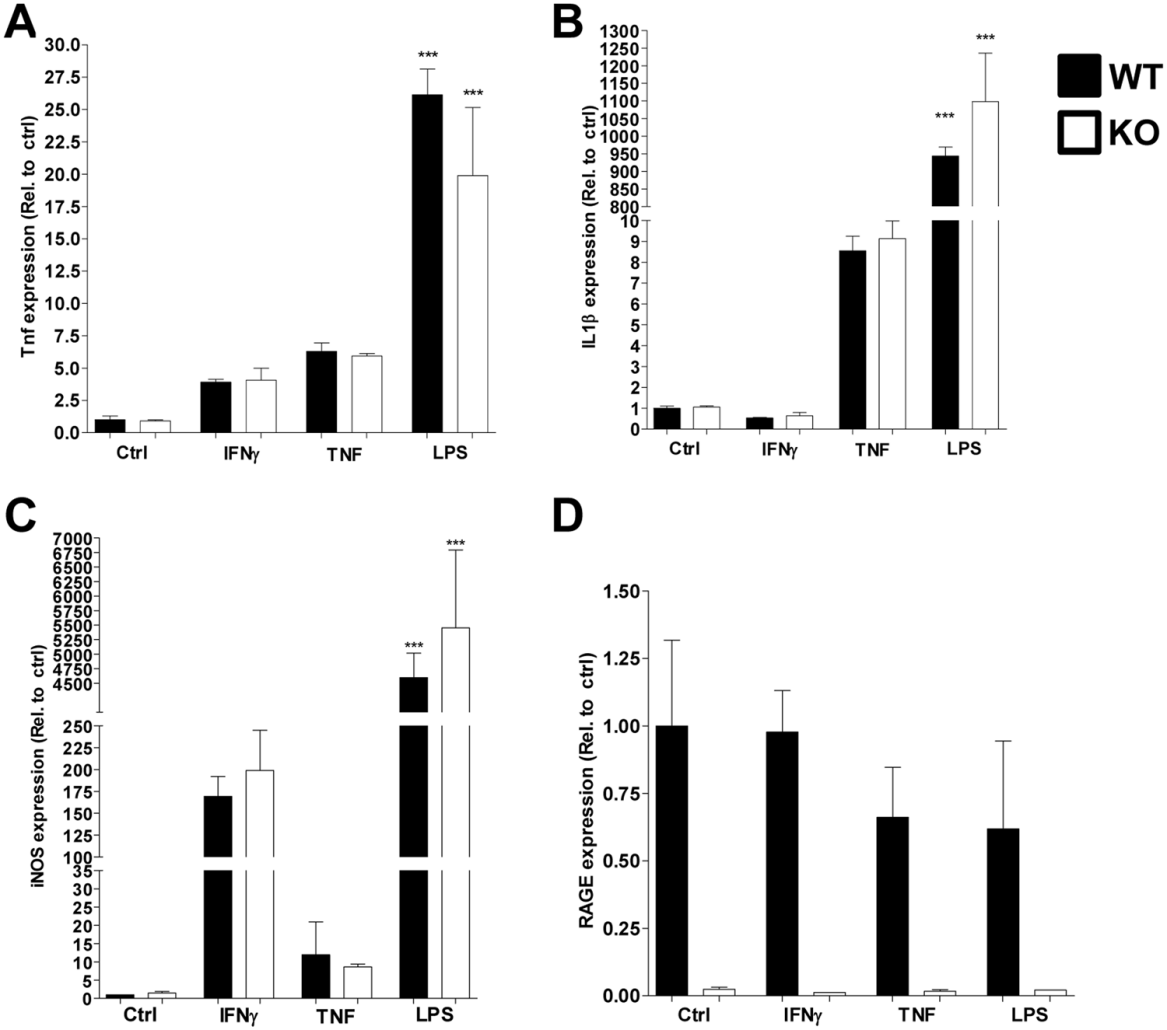
RAGE does not contribute to inflammatory signalling in macrophages. (**A**–**D**) Gene expression levels of Tnf (**A**), IL1β (**B**), iNOS (**C**) and RAGE (**D**) after stimulation of control (Ldlr^−/−^) or RAGE^−/−^ (Ldlr^−/−^RAGE^−/−^) BMDMs with IFNɣ, TNF or LPS for two hours. All data are means ± SEM. ***P < 0.001 vs control. n = 3.

As we were unable to detect any differences between control and RAGE-deficient mice in NASH development, we investigated the contribution of RAGE to general macrophage inflammation. When stimulating both control (Ldlr^−/−^ mice derived) and RAGE-deficient (Ldlr^−/−^RAGE^−/−^ mice derived) macrophages with IFNɣ, TNF or LPS for 2 hours, expression of *Tnf* increased in a RAGE-independent manner (Fig. 4A). *IL1β* only increased after stimulation with TNF and LPS, but not IFNɣ and this increase was also comparable between control and RAGE-deficient macrophages (Fig. 4B). Lastly, inducible nitric oxide synthase (*iNOS*) expression was increased by all stimuli, but the increase due to TNF was only minor compared to IFNɣ or LPS stimulation. There was no difference between control and RAGE^−/−^ derived macrophages (Fig. 4C). RAGE expression was unaffected by all stimuli and was nearly undetectable in RAGE-deficient BMDMs (Fig. 4D).

### RAGE deficiency does not affect circulating and hepatic free AGEs

Previous research showed RAGE-mediated trapping of CML in vAT of obese mice, which could induce inflammatory cytokine secretion^18^. To explore whether RAGE deficiency affects hepatic AGE trapping, we measured circulating and hepatic α-dicarbonyls and free AGE levels after 12 weeks of WTD feeding. Circulating and hepatic levels of MGO, GO and 3-DG were all comparable between control and RAGE-deficient mice. Plasma and liver free AGE levels were also unaffected by RAGE (Table 1). These results suggest RAGE-mediated trapping of CML, or other AGEs, does not occur in the liver. There are several other receptors associated with binding to AGEs, *i.e*. dolichyl-diphosphooligosaccharide–protein glycosyltransferase (DDOST), CD36 and Galectin-3. We determined the gene expression levels of these receptors in the liver. *Ddost* and *Galectin-3* expression were comparable between control and RAGE-deficient mice. Interestingly, *CD36* expression was upregulated in RAGE-deficient mice (Suppl. Fig. 5). Next, we determined AGE levels in the vAT of our mice to investigate if AGE trapping occurs in our model or is restricted to vAT during obesity. No difference in CML or MG-H1 was observed between control and RAGE-deficient mice in our hypercholesteraemic mice (Suppl. Table 5).

**Table 1:** Circulating and hepatic α-dicarbonyls and free AGEs are not affected by RAGE.

	WT (Mean ± SEM)	KO (Mean ± SEM)
Plasma MGO (nmol/L)	1267 ± 238	853 ± 125
Plasma GO (nmol/L)	4953 ± 424	5507 ± 266
Plasma 3-DG (nmol/L)	2595 ± 147	2757 ± 176
Hepatic MGO (nmol/g)	2987 ± 169	2930 ± 200
Hepatic GO (nmol/g)	4676 ± 380	4923 ± 477
Hepatic 3-DG (nmol/g)	11336 ± 1553	9980 ± 1404
Plasma CML (nmol/L)	86,6 ± 6,5	74,6 ± 5,6
Plasma CEL (nmol/L)	21,5 ± 1,1	22,7 ± 0,9
Plasma MG-H1 (nmol/L)	8,4 ± 1,7	8,9 ± 1,2
Hepatic CML (nmol/g)	2,53 ± 0,11	2,49 ± 0,08
Hepatic CEL(nmol/g)	5,28 ± 0,44	4,49 ± 0,17
Hepatic MG-H1 (nmol/g)	0,72 ± 0,06	0,63 ± 0,06

The α-dicarbonyls MGO, GO and 3-DG measured in plasma and liver after 12 weeks of WTD feeding. In addition, the free CML, CEL and MG-H1 levels in the circulation and the liver after 12 weeks of WTD. n = 11–12.

## Discussion

Our study showed no key role of RAGE in the development of NASH or atherosclerosis in hyperlipidemic mice. No effects of RAGE deficiency on systemic or hepatic inflammation or on hepatic steatosis were observed. In addition, RAGE deficiency did not impact inflammatory gene expression in the adipose tissue or aorta and there was no effect on atherosclerotic plaque size or phenotype. Lastly, RAGE deficiency did not affect circulating or hepatic levels of AGEs.

RAGE-deficient mice exhibited reduced weight gain, likely as a result of a slight difference in visceral fat expansion. Further examination of the vAT revealed no clear effects on adipocyte cell size or lipid metabolism. Potentially, adipocyte numbers are altered as Monden *et al*. suggested that RAGE may have a role in adipogenesis. Blocking RAGE in an *in vitro* model using 3T3-L1 adipocytes resulted in larger adipocytes and accelerated adipocytic differentiation, but we were unable to evaluate this *in vivo*^32^. Ueno *et al*. also observed reduced weight gain and reduced visceral fat expansion in RAGE-deficient mice, but in a different model of atherosclerosis development (apoE^−/−^)^33^. In addition, Song *et al*. showed this effect in high fat diet-induced obesity in RAGE-deficient mice and linked it to increased energy expenditure and a reduction of CD11c^+^ macrophages in vAT^34^. Moreover, a decrease in adipocyte size and AT inflammation was observed in the RAGE-deficient obese mice in their study. We did not observe such a clear effect of RAGE deficiency on adipocyte size or adipose tissue inflammation, possibly due to the differences between mouse models, such as the diets. The high fat diet used by Song *et al*. consists of 60% lard designed to greatly expand adipose tissue mass and cause recruitment of macrophages causing inflammation^34,35^. In contrast, we used WTD with 21% fat (milk butter) and 0.2% cholesterol, which induces mild obesity^20,36^. Therefore, any effects of RAGE on adipocyte size or adipose tissue inflammation could differ between these models depending on the diets.

RAGE deficiency did not affect WTD-induced monocytosis. This is consistent with a study performed by Nagareddy *et al*. showing no effects on leukocytosis and also specifically monocytosis after transplanting RAGE^−/−^ bone marrow to obese mice^37^. Without a clear effect on hyperlipidemia-associated monocytosis, a role for RAGE on monocyte-derived macrophages in tissues such as the liver is less likely.

RAGE deficiency did not affect WTD-induced hepatic steatosis, neutrophil recruitment or macrophage accumulation. Comparable levels of steatosis were expected as no effects of RAGE on hepatic cholesterol or triglycerides storage have been described and RAGE has mainly been described as a protein important in inflammation^38^. Nonetheless, Daffu *et al*. did report a role for RAGE in macrophage cholesterol efflux via suppression of ABCG-1, leaving room for speculation on roles of RAGE outside of inflammatory signalling^39^.

Unexpectedly, we did not observe any effects of RAGE on hepatic inflammation at two different time points (3 and 12 weeks) and NASH development was unaltered by RAGE deficiency. Previous studies revealed a potential role of RAGE in several other immune-driven liver pathologies such as acetaminophen-induced liver injury, ischemic liver injury and cholestatic liver disease^40–42^. Furthermore, our group and others showed an accumulation of AGEs and the presence of RAGE in murine and human livers and associations between serum AGE levels and liver pathology have been described^9,43,44^. In addition, Leung *et al*. reported exacerbation of fatty liver disease including inflammatory markers when feeding rats a high AGE diet and these effects could be RAGE-dependent^44^. In spite of this previous research suggesting a potential role of RAGE in NAFLD and in particular NASH, we did not find any clear effects of RAGE deficiency on NASH development in our model. However, there is a major distinction between the inflammatory responses when comparing models of liver injury with a model causing hepatic inflammation due to dietary cholesterol. In the first, major damage-associated molecular patterns (DAMPs) are released upon cell injury and death, triggering a strong and fast inflammatory response^45^. HMGB1, an important DAMP released upon cell damage, is known to bind and activate RAGE signalling and consequently trigger inflammation^46,47^. In the WTD-feeding (rich in cholesterol) model, hepatocytes are sensitized to TNF and FAS by cholesterol loading of the mitochondria^48^. In addition, Kupffer cells take up cholesterol and trap oxidized LDL triggering an immune response^20,49^. Therefore, in WTD-induced NASH, DAMPs might be of less importance and are likely not released in high amounts due to a lack of substantial liver damage in this model^19^. Therefore, any role of RAGE in our model would likely be via effects of AGEs on RAGE. In agreement, Leung *et al*. showed no effects of RAGE deficiency on NAFLD unless hepatic AGEs were strongly increased by feeding mice a baked diet which contained an increased amount of CML, a proven ligand of RAGE^50^. This could suggest that dietary AGEs contribute more to hepatic AGE levels than endogenous formation stimulated by hepatic lipid accumulation and inflammation.

We previously reported involvement of RAGE in trapping CML in vAT of obese mice resulting in inflammation and in reduced circulating CML levels^18^. In the current study using WTD-fed mice, circulating, hepatic and vAT CML levels were unaffected by RAGE deficiency. These observations indicate that CML trapping does not occur in the liver during NASH development, possibly explaining why we find no role for RAGE in the development of hepatic inflammation. Interestingly, our data suggest that the RAGE-dependent CML trapping and subsequent inflammation in adipose tissue does not play a major role in our model. It is possible that CML trapping by RAGE can only be observed in adipose tissue during the development of more severe obesity^18^. Potentially, RAGE needs to be activated by adipose tissue inflammation to trap CML. Obesity caused by high fat diet feeding or genetic mutation, *i.e*. the DB/DB mouse in our previous study, triggers strong adipose tissue inflammation^18^. The Western diet used in current study likely did not strongly induce inflammation in the adipose tissue. In line, we did not observe a difference in AT inflammation between control and RAGE-deficient mice, while this was previously observed in the DB/DB obese mice. Remarkably, a difference in body weight in the DB/DB mice between controls and RAGE-deficient mice was not observed in our previous study. Together, these data illustrate that the function or activation status of RAGE might be different in diverse metabolic and inflammatory states. Another potential explanation for comparable AGE levels in spite of the absence of RAGE might be a compensatory mechanism by other AGE-receptors. In our study, we determined an elevated expression of the AGE-receptor CD36. CD36 can bind and take up AGEs and has been associated with NF-κB activation and consequently inflammation^51,52^.

RAGE-deficient mice displayed a twofold higher expression level of Glo-1 in the liver corresponding with an earlier finding by Reiniger *et al*. showing a higher level of Glo-1 in the kidney of RAGE-deficient diabetic mice^53^. This might indicate that RAGE negatively controls Glo-1 expression. Glo-1 is the rate-limiting enzyme in the detoxification of the major AGE precursor MGO. However, the observed higher levels of Glo-1 expression did not impact hepatic MGO levels in our study, likely due to unchanged Glo-1 activity in the RAGE-deficient mice. Glo-1 activity is dependent on the availability of glutathione, an anti-oxidant^31^. It is possible that oxidative stress caused by hepatic inflammation leads to glutathione depletion. Therefore, the glyoxalase system might not have been able to impact AGE levels despite an increased expression of Glo-1. In line, the elevated Glo-1 expression induced by RAGE deficiency as observed in the current study did not impact atherosclerosis. Previous work by our group also showed no effects of Glo-1 overexpression on atherosclerosis^31^. In contrast, Reiniger *et al*. showed a decrease of MGO levels in the kidney of RAGE-deficient mice, but the observed decrease by Reiniger *et al*. was only observed in diabetic mice and not in their non-diabetic control mice^53^. Moreover, Glo-1 activity was not determined in their study, but might have been increased in their RAGE-deficient mice.

Corresponding with our observation that monocytosis and hepatic inflammation were unaffected by RAGE deficiency, we did not observe an effect of RAGE on atherosclerotic plaque size or plaque inflammatory gene expression. Previous research did report effects of RAGE on atherosclerosis, mainly in diabetes-induced atherosclerosis in which the pathogenesis is different from the WTD-driven atherosclerosis^54^. However, Sun *et al*. investigated the effects of RAGE deficiency on atherosclerosis in a similar model, the Ldlr^−/−^ mouse fed a high fat diet containing cholesterol^17^. A main difference between the used diets is cholesterol levels (0.15% in their study instead of the 0.20% in our study) and fat content (36.2% in their study and not 21% as in our study). Alternatively, discrepancies might also be explained by other diet components (vitamins, minerals, etc.), genetic background, health status and even microflora.

In the Ldlr^−/−^ mouse fed a WTD, the model for NASH used in this study, hepatic steatosis and inflammation occurs rapidly. Other, mainly late stage aspects of NASH, such as fibrosis and liver damage characterised by hepatocyte ballooning are mild^19,20^. When liver damage does occur in NASH and DAMPs *e.g.* HMGB1 are released, RAGE could have a superior role to play in aggravating hepatic inflammation.

In conclusion, RAGE does not seem to play a major role in the development of NASH in hyperlipidemic mice. However, we cannot rule out that AGEs, via other mechanisms, play a role in NAFLD. Their effect on the inflammatory aspect of NAFLD needs to be further investigated, preferably by inhibiting AGE formation or altering dietary AGE intake.

## Electronic Supplementary Material

Supplementary file

## Data Availability

The datasets generated during and/or analysed during the current study are available from the corresponding author on reasonable request.
