# Supplementary material for: RAGE deficiency does not affect non-alcoholic steatohepatitis and atherosclerosis in Western type diet-fed Ldlr−/− mice
Source: Sci Rep. 2018 Oct 15;8:15256. doi: 10.1038/s41598-018-33661-y (PMC6189204; doi:10.1038/s41598-018-33661-y)
Supplement: Supplementary file 1 — Supplementary file [file 41598_2018_33661_MOESM1_ESM.pdf]

RAGE deficiency does not affect non-alcoholic steatohepatitis and atherosclerosis in Western type diet-fed Ldlr$^{-/-}$ mice

Mitchell Bijnen[1,2], Nicky Beelen[1,2], Suzan Wetzels[1,2,3], José van de Gaar[1,2], Maria Vroomen[1,2], Erwin Wijnands[2,4], Jean L. Scheijen[1,2], Marjo P.H. van de Waarenburg[1,2], Marion J. Gijbels[2,4,5,6], Jack P. Cleutjens[2,4], Erik A.L. Biessen[2,4], Coen D.A. Stehouwer[1,2], Casper G. Schalkwijk[1,2] and Kristiaan Wouters*[1,2]

[1] Dept. of Internal Medicine, MUMC, Maastricht, The Netherlands, [2] CARIM, MUMC, Maastricht, The Netherlands, [3] Dept. of Immunology and Biochemistry, Biomedical Research Institute, Hasselt University, Hasselt, Belgium, [4] Dept. of Pathology, MUMC, Maastricht, The Netherlands, [5] Dept. of Molecular Genetics, MUMC, Maastricht, The Netherlands, [6] Dept. of Medical Biochemistry, Experimental Vascular Biology, AMC, Amsterdam, The Netherlands.

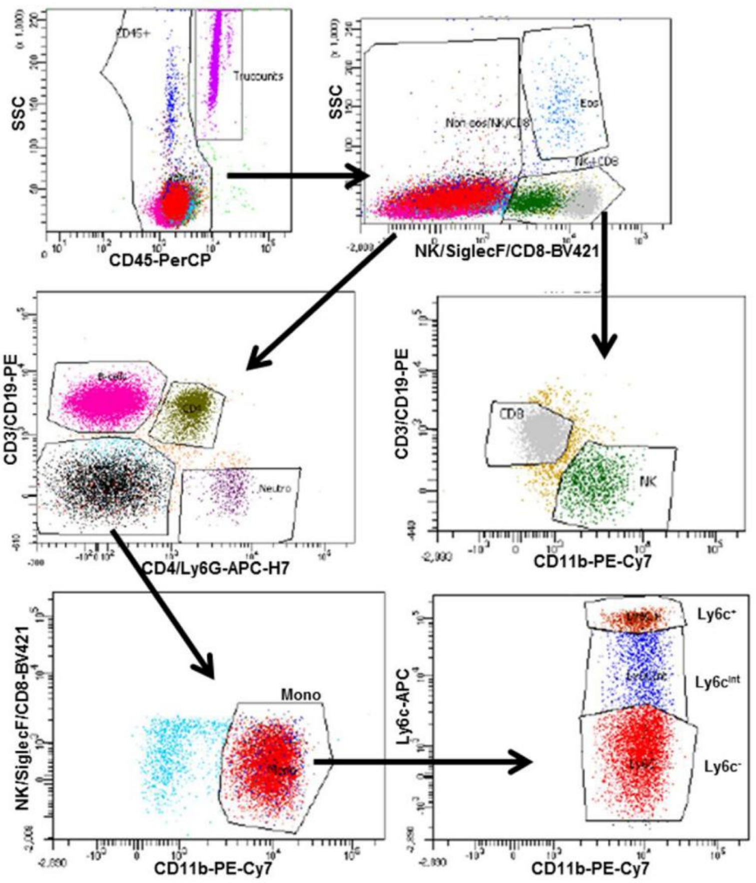

Suppl. Fig. 1. Gating strategy used for flow cytometry

FACS plots showing gating strategy used for circulating cells starting from CD45^{+} cells. First, eosinophils (SSC^{+}, SiglecF^{+}), NK (SSC^{-}, NK1.1^{+}) and CD8^{+} T-cells (SSC^{-}, CD8^{+}) were separated and further divided into CD8^{+} T-cells (CD3^{+}) and NK-cells (CD11b^{+}). All other cells were divided into B-cells (CD19^{+}, CD4^{-}, Ly6G^{-}), CD4^{+} cells (CD3^{+}, CD4^{+}), neutrophils (CD3^{-}, Ly6G^{+}) and remaining cells. Monocytes were separated from these remaining cells using CD11b before splitting all monocytes into Ly6c^{-} (patrolling monocytes), Ly6c^{int} and Ly6c^{+} (proinflammatory) monocytes based on their Ly6c expression. SSC, sidescatter.

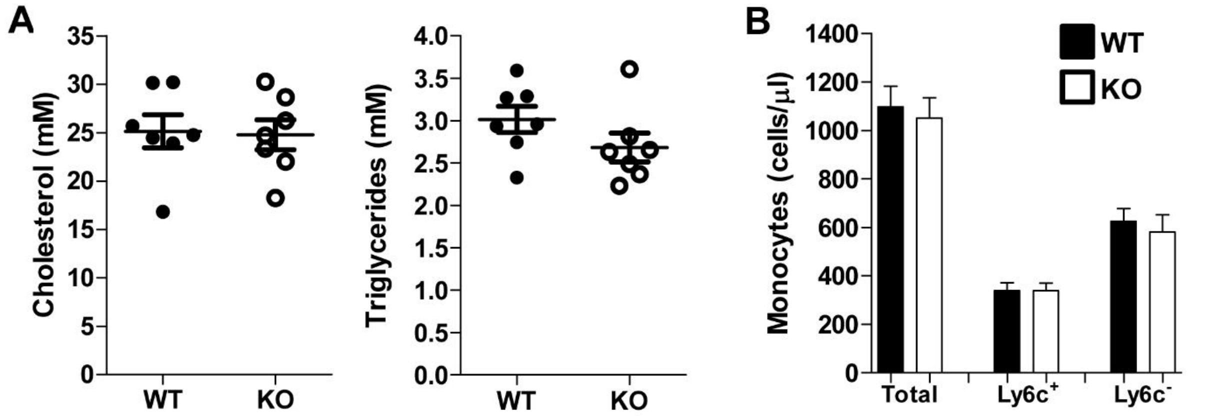

Suppl. Fig. 2. RAGE does not affect plasma lipid levels or monocytosis

(A) Plasma cholesterol and triglyceride levels after 3 weeks of WTD feeding. (B) Total circulating monocyte levels and subdivision in Ly6C^{+} and Ly6C^{-} monocytes measured by flow cytometry and presented as cells/µl after 3 weeks of WTD feeding. All data are means ± SEM. n = 7.

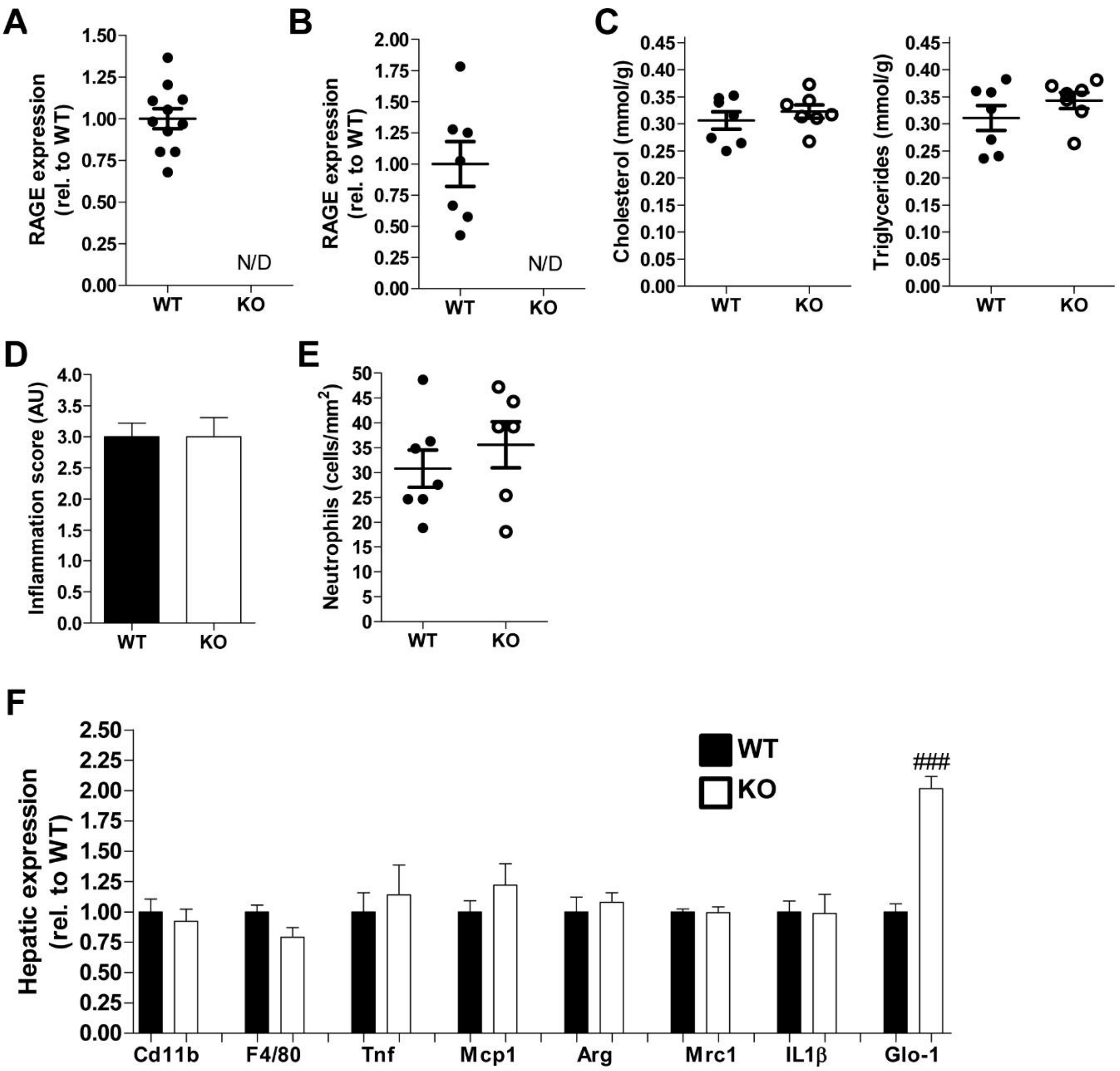

Suppl. Fig. 3. Hepatic steatosis, neutrophil infiltration and macrophage accumulation were unaffected by RAGE

(A, B) Hepatic RAGE expression levels after 12 (A; n = 11-12) and 3 weeks (B; n = 7) of WTD feeding. (C) Hepatic cholesterol and triglycerides levels after 3 weeks of WTD feeding. (D) Hepatic inflammation score (1, 2, 3 or 4 based on lobular inflammation and immune cell numbers) after 3 weeks of WTD feeding. (E) Quantification of neutrophils in the liver after 3 weeks of WTD. (F) Hepatic gene expression levels of inflammatory and immune cell specific markers after 3 weeks of WTD feeding. N/D = not detectable. All data are means ± SEM. ###P < 0.001 vs WT. n = 7.

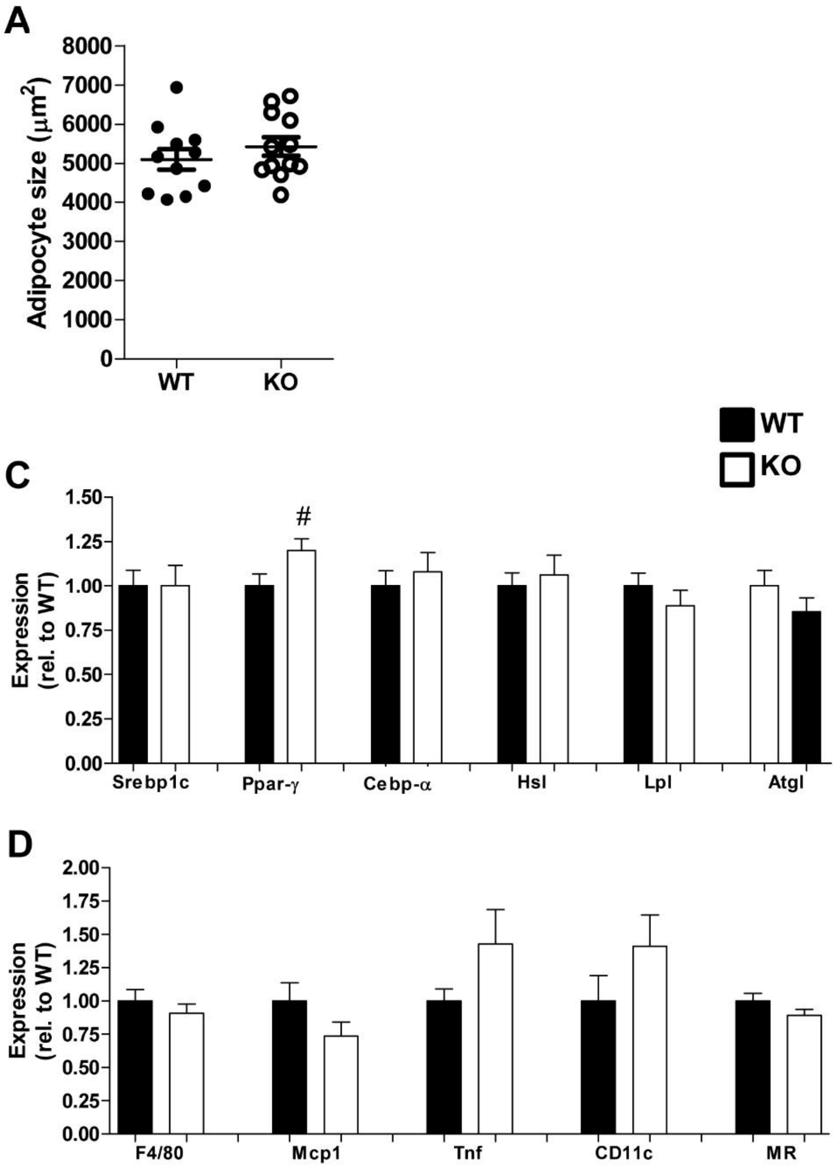

Suppl. Fig. 4. RAGE deficiency does not influence adipocyte size, lipid metabolism or adipose tissue inflammation

(A) Adipocyte cell size after 12 weeks of WTD feeding. (B) Lipolysis and adipogenesis related gene expression in the vAT. (C) Gene expression levels of inflammatory and immune cell specific markers in vAT after 12 weeks of WTD feeding. All data are means ± SEM. n = 11-12.

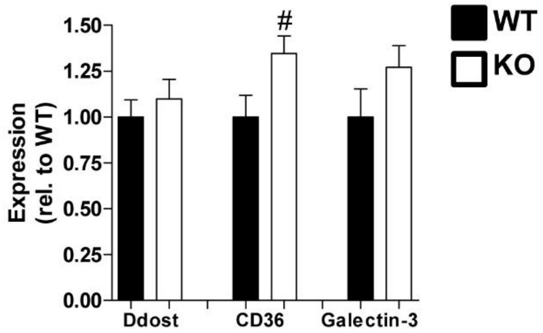

Suppl. Fig. 5. The absence of RAGE stimulates expression of CD36, but not of other AGE-receptors

Gene expression levels of the AGE-receptors DDOST, CD36 and Galectin-3 in the liver after 12 weeks of WTD feeding. All data are means ± SEM. n = 11-12.

Suppl. Table 1. Antibodies used for flow cytometry of circulating cells

Flow cytometry antibodies with fluorescent label and manufacturer.

Antibody	Label	Manufacturer
**Blood mix**		
CD16/32	-	eBioscience
CD45	PerCP	Biolegend
CD3	PE	eBioscience
NK1.1	BV421	Biolegend
LY6G	APC-H7	BD
CD11B	PE-Cy7	BD
LY6C	APC	Miltenyi
CD4	APC-H7	BD
CD8	eFluor450	eBioscience
CD19	PE	eBioscience
SIGLECF	BV421	BD

Suppl. Table 2. Primer sequences

Forward and reverse qPCR primer sequences of all measured genes.

Gene	FW primer	RV primer
**Mcp1**	TTAAAAACCTGGATCGGAACCAA	GCATTAGCTTCAGATTTACGGGT
**Tnf**	CATCTTCTCAAAATTCGAGTGACAA	TGGGAGTAGACAAGGTACAACCC
**F4/80**	GGATGTACAGATGGGGGATG	TCTGTGGTGTCAGTGCAGGT
**IL1β**	CTGCAGCTGGAGAGTGTGGAT	CACTTTGCTCTTGACTTCTATCTTGTTG
**Cd11b**	ATGGACGCTGATGGCAATACC	TCCCCATTCACGTCTCCCA
**Mrc1**	TGCCAAAAATTATTGATCCTGTAACT	CGCCGGCACCTATCACA
**Arg1**	CATGGGCAACCTGTGTCCTT	CGATGTCTTTGGCAGATATGCA
**Glo1**	ATGACGAGACTCAGAGTTACCACAA	TAGACATCAGGAACGGCAAATCC
**RAGE**	GCTCGAATCCTCCCCAATG	TCCCCTCATCGACAATTCCA
**Cd11c**	CTGGATAGCCTTTCTTCTGCTG	GCACACTGTGTCCGAACTCA
**IL-6**	TTCAACCAAGAGGTAAAAGATTTACATAA	CACTCCTTCTGTGACTCCAGCTT
**iNOS**	GCCCCTGGAAGTTTCTCTTC	TTCTGTGCTGTCCCAGTGAG
**Col1a1**	TGTTCAGCTTTGTGGACCTC	TCAAGCATACCTCGGGTTTC
**Srebp1c**	ACTTTTCCTTAACGTGGGCCT	ACTTTTCCTTAACGTGGGCCT
**Ppar-γ**	ATGATGGGAGAAGATAAAATCAAGTTC	CGGCTTCTACGGATCGAAAC
**Cebp-α**	GCGGGAACGCAACAACATC	GTCACTGGTCAACTCCAGCAC
**Hsl**	GAGGCCTTTGAGATGCCACT	AGATGAGCCTGGCTAGCACAG
**Lpl**	TGGCGTAGCAGGAAGTCTGA	TGCCTCCATTGGGATAAATGTC
**Atgl**	AGCATCTGCCAGTATCTGGTGAT	CACCTGCTCAGACAGTCTGGA
**Ddost**	CCCGGACAATCCCTTGGTTT	TGAGAATACCTCTGCGCACC
**Cd36**	GGAGCCATCTTTGAGCCTTCA	GAACCAAACTGAGGAATGGATCT
**Galectin-3**	CCCGCATGCTGATCACAATC	GGGGTTAAAGTGGAAGGCAA
**Cyclophilin**	TTCCTCCTTTCACAGAATTATTCCA	CCGCCAGTGCCATTATGG
**B2-Micro**	CTTTCTGGTGCTTGTCTCACTGA	GTATGTTCGGCTTCCCATTCTC

Suppl. Table 3. RAGE deficiency does not affect circulating immune cells

Levels of circulating T-cells, B-cells, neutrophils, NK-cells and eosinophils measured by flow cytometry in cells/µl after 12 weeks of WTD feeding. n = 11-12.

Cell type	WT (Mean ± SEM)	KO (Mean ± SEM)
All immune cells (cells/µl)	6268 ± 499	7309 ± 824
CD4 T-cells (cells/µl)	590 ± 53	731 ± 86
CD8 T-cells (cells/µl)	591 ± 37	625 ± 72
B-cells (cells/µl)	2744 ± 303	3741 ± 471
Neutrophils (cells/µl)	911 ± 289	1075 ± 245
NK-cells (cells/µl)	425 ± 77	296 ± 26
Eosinophils (cells/µl)	63,8 ± 11,2	109,3 ± 27,3

Suppl. Table 4. Circulating immune cells are not affected by RAGE

Levels of circulating T-cells, B-cells, neutrophils, NK-cells and eosinophils measured by flow cytometry in cells/µl after 3 weeks of WTD feeding. #P < 0.05 vs WT. n = 7.

Cell type	WT (Mean ± SEM)	KO (Mean ± SEM)
All immune cells (cells/µl)	10969 ± 393	12570 ± 723
CD4 T-cells (cells/µl)	1656 ± 124	1832 ± 139
CD8 T-cells (cells/µl)	1204 ± 79	1221 ± 76
B-cells (cells/µl)	5448 ± 213	6929 ± 456[#]
Neutrophils (cells/µl)	657 ± 70	684 ± 97
NK-cells (cells/µl)	257 ± 21	230 ± 18
Eosinophils (cells/µl)	138 ± 16	177 ± 20

Suppl. Table 5. Free AGE levels in the vAT are not affected by RAGE-deficiency

The CML, CEL and MG-H1 levels in the vAT after 12 weeks of WTD. n = 11-12. N/D = not detectable.

vAT AGE levels	WT (Mean ± SEM)	KO (Mean ± SEM)
CML (nmol/g)	78,7 ± 15,1	86,0 ± 15,2
CEL (nmol/g)	N/D	N/D
MG-H1 (nmol/g)	4,08 ± 0,64	3,84 ± 0,71
